# Assessment of Angiography-Based Renal Quantitative Flow Ratio Measurement in Patients with Atherosclerotic Renal Artery Stenosis

**DOI:** 10.1155/2024/4618868

**Published:** 2024-01-09

**Authors:** Xiang Huang, Xiao-Lan Li, Heng Zhou, Xiao-Mei Li

**Affiliations:** Department of Cardiology, Xiangyang No.1 People's Hospital, Hubei University of Medicine, Xiangyang 441000, Hubei, China

## Abstract

**Background:**

Quantitative flow ratio (QFR) is an angiography-based fractional flow reserve measurement without pressure wire or induction of hyperemia. A recent innovation that uses combined geometrical data and hemodynamic boundary conditions to measure QFR from a single angiographic view has shown the potential to measure QFR of the renal artery-renal QFR (rQFR).

**Objective:**

The aim of this pilot study was to assess the feasibility of rQFR measurement and the contribution of rQFR in selecting patients with atherosclerotic renal artery stenosis (ARAS) undergoing revascularization.

**Methods:**

This retrospective trial enrolled patients who had ARAS (50-90%) and hypertension. The enrolled patients were treated by optimal antihypertensive medication or revascularization, respectively, and the therapeutic strategies were based on rFFR measurement and/or clinical feature.

**Results:**

A total of 55 patients underwent rQFR measurement. Among the enrolled patients, 18 underwent optimal antihypertensive medication and 37 underwent revascularization, 19 patients in whom rQFR and rFFR were both assessed. During the 180-day follow-up, 25 patients saw an improvement in their blood pressure among the 37 patients that underwent revascularization. ROC analysis revealed that rQFR had a high diagnostic accuracy for predicting blood pressure improvement (AUC_rQFR_ = 0.932, 95% CI 0.798-0.998). The ideal cut-off value of rQFR for predicting blood pressure improvement after revascularization is ≤0.72 (sensitivity: 72.00%, specificity: 100%). The paired *t* test and Bland–Altman analyses demonstrated good agreement between rQFR and rFFR (*t* = 1.887, 95% CI -0.021 to 0.001, 95% limits of agreement: -0.035 to 0.055, *p* = 0.075). The Spearman correlation test reveals that there was a significant positive correlation between rQFR and rFFR (*r* = 0.952, 95% CI 0.874 to 0.982, *p* < 0.001).

**Conclusion:**

The rQFR has the potential to enhance the ability of angiography to detect functionally significant renal artery stenosis during angiography and to produce results that are comparable to invasive hemodynamic assessment.

## 1. Introduction

Percutaneous transluminal renal angioplasty with stent placement (PTRAS) has emerged as the preferred technique to treat atherosclerotic renal artery stenosis (ARAS) with unmanageable hypertension or progressive renal failure [1]. Despite prospective and randomized trials like the ASTRAL (Angioplasty and Stent for Renal Artery Lesions) and CORAL (Cardiovascular Outcomes in Renal Atherosclerotic Lesions) trials failing to demonstrate the advantages of PTRAS over medical therapy on preserving renal function, controlling blood pressure, or improving cardiovascular outcome in patients with ARAS, a considerable range of smaller clinical studies have shown the opposite result [2]. A number of researchers pointed out that ASTRAL and CORAL both have the restriction of not including certain subsets of patients who have high-risk clinical manifestations, such as episodic pulmonary edema, rapidly progressing renal failure, and resistant hypertension, which are more likely to benefit from revascularization [3]. Instead, these two trials involved a large number of patients with moderate to severe chronic renal failure, who might have enrolled in the studies too late to benefit from PTRAS [4]. The variable response to revascularization is likely caused by the fact that the selection of renal arteries suitable for PTRAS is based on anatomic grading of the stenosis using angiography rather than functional assessment, in addition to differences in the clinical characteristics of the patients [5].

To optimal patient selection for PTRAS more effectively, pressure and/or flow measurements performed during hyperemia may be crucial for determining the true hemodynamic significance of the renal artery stenoses. Renal fractional flow reserve (rFFR), defined as the ratio of renal artery distal pressure (Pd) to proximal aortic pressure (Pa) during hyperemic conditions (rFFR = Pd/Pa), was associated with a significant increase in blood pressure following revascularization, according to post hoc analyses of related research [4]. Prior researches have suggested that moderate ARAS (50-70%) with rFFR < 0.8 is considered an indication for renal artery revascularization [6, 7]. Nonetheless, the use of pressure wire-based renal artery physiological assessment has not yet become part of the routine in catheterization laboratories for several reasons, including the need to advance a pressure wire into the renal artery to interrogate the stenosis, the cost of the wire, and the controversial of renal hyperemic agent selection.

As an alternative to invasive hemodynamic assessment without the use of a pressure wire or the induction of hyperemia, quantitative flow ratio (QFR), an angiographically derived FFR measurement, was created [8]. Previous investigations have shown that at the level of coronary circulation, QFR and FFR measures have a good agreement [9]. A recent innovation that is based on artificial intelligence and the Murray bifurcation fractal law to measure QFR from a single angiographic view has shown the potential to measure QFR of the renal artery [10].

In this study, we propose that renal QFR (rQFR) and rFFR might have similar diagnostic value in patients with ARAS. As a result, the purpose of this pilot study was to evaluate the feasibility of rQFR measurement and the contribution of rQFR in selecting patients with ARAS undergoing PTRAS.

## 2. Methods

### 2.1. Study Design and Populations

This investigation retrospected consecutive patients who had ARAS and hypertension over time at Xiangyang No.1 People's Hospital. The following criteria were used for inclusion: unilateral stenosis, diameter stenosis rate of 50% to 90%, and lesion lengths estimated visually ≥2 mm. The following conditions were considered to be exclusion criteria: severe ostial stenosis, severe vessel overlap or tortuosity at the stenotic segments, nonatherosclerotic renal artery stenosis, bilateral lesions, tandem lesions, low-quality angiographic images preventing contour detection, incomplete record of clinical or accessory examinations, and failure to be followed up.

The therapeutic strategies of revascularization or optimized antihypertensive medications were based on rFFR measurement or clinical feature. Revascularization was performed in patients with rFFR < 0.8 or high-risk clinical presentations, including episodic pulmonary edema, rapidly progressing renal failure, and resistant hypertension, which more likely to benefit from revascularization. For the rest of the patients, optimal antihypertensive medication treatment had been used.

The Xiangyang No.1 People's Hospital Ethics Committee accepted the study procedure, which complies with the Declaration of Helsinki's ethical standards for medical research involving human beings.

### 2.2. Analysis of Quantitative Renal Angiographic (QRA) and Renal Quantitative Flow Ratio (rQFR)

Invasive renal artery angiography and PTRAS were performed according to relevant guideline recommendations. Two skilled and independent analysts used QFR software (Angio-PLUS, Pulse Medical Imaging Technology, Shanghai Co., Ltd., Shanghai, China) to analyze all enrolled patients' angiographic images, which were captured at a rate of 15 frames per second by radiography systems (GE Innova 3100-IQ, GE Healthcare, Wauwatosa, Wisconsin, USA). The clinical studies and the follow-up findings were hidden from the analysts. The detailed computational algorithms of rQFR can be summarized as follows. (1) Firstly, a convolutional neural network-based artificial intelligence method was used to autonomously identify the luminal margins (contours) of the target vessels [11]. (2) Secondly, the Murray bifurcation fractal law was used to recreate a step-down reference vessel diameter function [10]. (3) The pressure drop caused by stenosis was then computed using fluid dynamics equations after obtaining the geometrical morphology of the stenosis, under the assumptions that the blood density is 1060 kg/m^3^ and the blood viscosity is 0.0035 kg/(m/s) [12]. For the computation of rQFR, the contrast flow model combining contrast flow velocity based on the frame count approach is applied. (4) The quantitative renal angiography (QRA) statistics (percent diameter stenosis (DS percent)) and the rQFR of the target vessel were finally made available by the software.

The capture of renal artery angiograms could not be carried out using an optimal technique for rQFR analysis due to the retrospective nature of the investigation. The contrast agent filling of fractional angiograms may not be satisfactory, thus necessitating manual contour correction during rQFR analysis.

### 2.3. rFFR Assessment

Before deciding to perform PTRAS for ARAS patients with diameter stenosis rates between 50% and 70% by visual judgment after selective renal angiography, rFFR was measured. Use a 6Fr JR4 guide catheter (Launcher™, Medtronic Inc., Minneapolis, Minnesota, USA) to perform selective cannulation of the right or left renal artery via femoral artery approach. A 0.014-inch pressure wire (Certus™, St. Jude Medical Inc., St. Paul, Minnesota, USA) was inserted into the guiding catheter after calibration. The guide catheter and pressure wire were used to simultaneously measure the aorta's pressures while the guide catheter was detached from the renal artery. Then, the height of the fluid-filled column was then changed to meet the pressure wire (i.e., equalization of the system). Subsequently, the pressure wire was then brought closer to the distal end of the renal artery. Simultaneous pressure measurements were obtained in the aorta (aortic pressure, Pa) from the distal end of the guide catheter and in the distal to the lesion (renal distal pressure, Pd) from the pressure wire. While the guide catheter was disengaged from the right renal artery, these readings were synchronously taken from the pressure wire and guide catheter. After papaverine-induced maximum hyperemia, which was achieved by injecting 30 mg of papaverine diluted in saline via the guide catheter to generate hyperemia, we measured the rFFR (defined by the ratio of Pd/Pa during maximum hyperemia) [13].

### 2.4. Follow-Up Evaluation

Office blood pressure was observed from all enrolled patients at 30, 90, and 180 days following discharged from the hospital. During the follow-up process, if deemed necessary, the antihypertensive medications of all enrolled patients were adjusted by a hypertension specialist based on home blood pressure monitoring as well as office blood pressure measurements. Detailed documentation was maintained regarding the types of antihypertensive medications prescribed to each enrolled patient and their corresponding office blood pressure readings. Following the procedure, renal duplex ultrasound scanning was done at 30 and 180 days.

### 2.5. Definitions

Diastolic blood pressure (DBP) < 90 mmHg and systolic blood pressure (SBP) < 140 mmHg while taking the same or fewer antihypertensive medications, or a decrease in office DBP of at least 15 mmHg while taking the same or decreased number antihypertensive medications, were considered improvements in blood pressure. When there was no change in blood pressure or when the conditions for improvement were not met, it was considered that blood pressure did not improve. If patients completed the requirements for blood pressure improvement within 90 days following discharged from the hospital, they were classified as responders; if not, they were classified as nonresponders [14].

### 2.6. Statistical Analysis

The research data were routinely tested for homogeneity of variance and normality distribution. Normally distributed quantitative data were expressed as mean ± standard deviation (SD) and statistical compared between groups using Student's *t* tests. Non-normal distribution quantitative data were expressed as median with interquartile range and statistical compared between groups using the Wilcoxon rank-sum test. Categorical variables are condensed as frequencies and proportions, compared between groups using Pearson's chi-square or Fisher's exact test. Repeated analyses were carried out by two independent analysts to assess the interobserver and intraobserver reliabilities in the rQFR analyses, and the intraclass correlation coefficient (ICC) was employed to assess the reliability of the repeated data. Using the area under the curve (AUC) and 95% confidence interval (CI) of the receiver operating characteristic (ROC) curve, the predictive value of rQFR for blood pressure improvement in ARAS patients with hypertension who underwent successful PTRAS was examined. The ideal cut-off value for predicting blood pressure improvement was found to be the rQFR value with the highest Youden index (sensitivity + specificity − 1). We used DeLong tests to assess the diagnostic effectiveness of QRA, rQFR, and QRA combined with rQFR. Correlation and agreement between rQFR and rFFR were determined by the Spearman correlation test and Bland–Altman analyses. The difference is considered statistically significant if the two-sided *p* value is less than 0.05. GraphPad Prism (version 8.3.0, GraphPad Software, San Diego, California, USA), SPSS (version 27.0, IBM Corp, Armonk, NY, USA), and MedCalc (version 20.0.1, MedCalc Software, Ostend, Belgium) were used for the statistical analysis.

## 3. Results

### 3.1. Patient Characteristics

**Figure 1:**
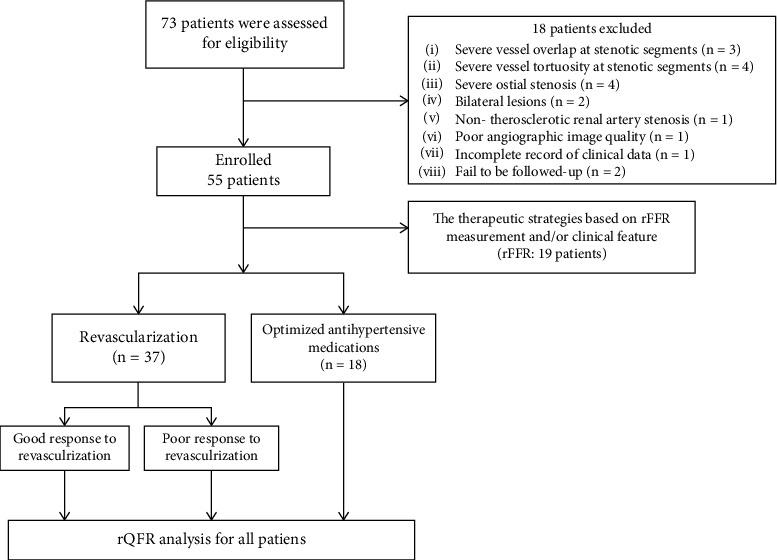
Study flowchart. A total of 55 consecutive patients were included in our study, 37 patients received stent revascularization, and 18 patients underwent optimized antihypertensive medications. rQFR and rFFR were both assessed in 19 vessels.

A total of 73 consecutive patients with hypertension and ARAS received treatment between January 2020 and January 2022 at Xiangyang No.1 People's Hospital. A total of 55 patients met the inclusion and exclusion criteria and had performed rQFR measurement. Among the enrolled 55 patients, 18 underwent optimal antihypertensive medication and 37 underwent PTRAS. Additionally, assessments of rQFR and rFFR were simultaneously conducted on 19 vessels from 19 patients.  Figure 1 depicts the study's flow, and Table 1 lists the clinical data of patients who underwent optimal antihypertensive medication and PTRAS.

### 3.2. Diagnostic Performance of rQFR and QRA

rQFR and QRA analyses of target renal arteries were performed on all enrolled patients. Investigations into the relationship between rQFR and blood pressure improvement after PTRAS were made. All patients received the necessary clinical follow-up. There were no complications found during subsequent hospital visits or the 180-day follow-up. Among the 37 patients that underwent PTRAS, 25 patients saw an improvement in their blood pressure (responders), while 12 patients did not see such an improvement (nonresponders). The intra- and interobserver reliabilities of rQFR were proved to be excellent (ICC = 0.997 and 0.998). Responders exhibit elevated levels of direct renin concentration, a reduced application of diuretics during the follow-up period, and these differences are statistically significant.  Table 2 lists the clinical data of responders and nonresponders.

**Figure 2:**
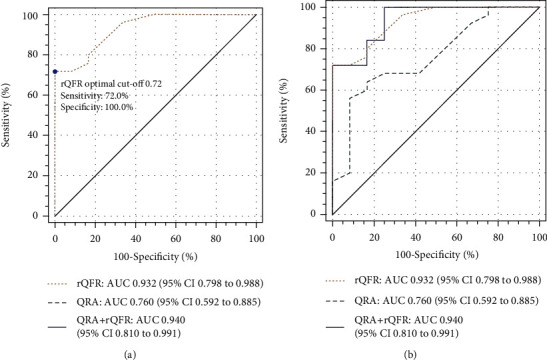
Comparison of diagnostic performance between rQFR and QRA by ROC analyses. (a) ROC curve shows optimal rQFR cut-off value (purple dot) for predicting blood pressure improvement after PTRAS. (b) Superiority of rQFR combined with QRA (blue line) compared to rQFR (orange line) or QRA (green line) in predicting blood pressure improvement after PTRAS.

ROC analysis revealed that rQFR had a high diagnostic accuracy for predicting blood pressure improvement (AUC_rQFR_ = 0.932, 95% CI 0.798-0.998, *p* < 0.001). The optimal cut-off value of rQFR for predicting blood pressure improvement after PTRAS is ≤0.72 (sensitivity: 72.00%, specificity: 100%).  Figure 2(a) depicts the ROC curve for the prediction of blood pressure improvement using rQFR. The use of rQFR to predict blood pressure improvement following PTRAS was significantly superior to QRA (AUC_rQFR_ = 0.932, AUC_QRA_ = 0.760, *p* = 0.0214). When rQFR and QRA were combined, the diagnostic efficiency got a further improvement (AUC_rQFR+QRA_ = 0.94, 95% CI 0.810-0.991). However, combined diagnosis had not demonstrated a statistically significant advantage over rQFR (AUC_rQFR+QRA_ = 0.94, AUC_rQFR_ = 0.932, *p* = 0.425). Comparisons are shown in Figure 2(b).

### 3.3. Correlation and Consistency between rQFR and rFFR

**Figure 3:**
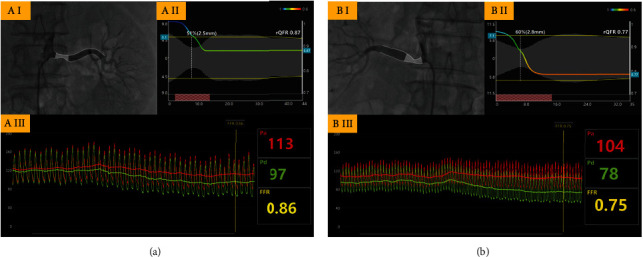
Representative examples of rQFR and rFFR assessments in our study. (a) A 63-year-old male was admitted due to hypertension with mild renal insufficiency. Due to renal artery duplex scanning which revealed that the resistance index of the left renal artery elevated to 0.81 (normal: 0.055-0.70), we performed selective renal angiography. QFR software automatic identified the stenosis in the proximal portion of the left renal artery (I). The QFR software analysis shows that diameter stenosis rate was 51%, minimal lumen diameter was 2.5 mm, reference vessel diameter was 5.1 mm, and rQFR was computed as 0.87 (II), while the invasive rFFR measured by pressure wire was 0.86 (III). (b) A 53-year-old female with resistant hypertension and unilateral renal atrophy underwent renal angiography. QFR software also automatically identified the stenosis in the proximal portion of the right renal artery (I). The QFR software analysis shows that diameter stenosis rate was 60%, reference vessel diameter was 7.1 mm, minimal lumen diameter was 2.8 mm, and rQFR was computed as 0.77 with a remarkable pressure drop (II), while the invasive rFFR measured by pressure wire was 0.75 (III).

**Figure 4:**
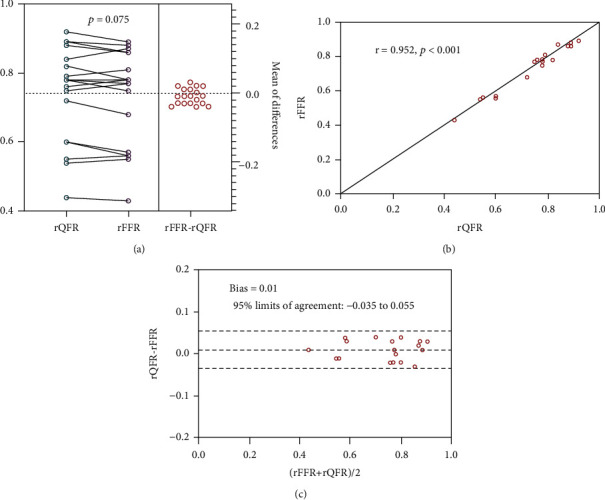
Agreement and correlation between rQFR and rFFR. Excellent correlation and agreement were demonstrated between rQFR and rFFR ((a) paired *t* test, (b) Spearman's correlation test, and (c) Bland–Altman analyses).

We compared rQFR and rFFR to further verify this method in lesions with hemodynamic relevance based on the extremely strong diagnostic effectiveness of rQFR analysis in predicting blood pressure improvement following PTRAS. A total of 19 vessels received both rQFR and rFFR measurement. Two representative rQFR and rFFR assessment examples from this study are shown in Figure 3. The paired *t* test and Bland–Altman analyses demonstrated good agreement between rQFR and rFFR (*t* = 1.887, 95% CI -0.021 to 0.001, 95% limits of agreement: -0.035 to 0.055, *p* = 0.075). The Spearman correlation test reveals that there was a significant positive correlation between rQFR and rFFR (*r* = 0.952, 95% CI 0.874 to 0.982, *p* < 0.001). A graphical representation of this comparison is shown in Figure 4.

## 4. Discussion

We believe that this is the first study to evaluate the feasibility of using rQFR, an angiography-based method for quick assessment of rFFR without the need of pressure wires or induction of hyperemia, to detect hemodynamically significant renal artery stenosis. Our data revealed that in a patient population with sequential enrollment, rQFR achieved comparable results to the invasive rFFR. rQFR demonstrated superior diagnostic performance in identifying the stenosis that actually caused hemodynamic disturbance, which may benefit from PTRAS, in comparison to QRA assessment. For patients with rQFR < 0.72, blood pressure improvement following PTRAS is more likely.

In previous studies, QFR measurement was relied on the 3-dimensional (3D) geometry reconstruction of two angiographic images acquired at different angles ≥25°, which extremely restricts its applications in peripheral vascular interventional therapy [8, 15]. However, in our study, the novel QFR calculation was based on a single plane angiographic images and acquired comparable diagnostic performance, which depend on step-down reference vessel diameter function reconstructed from the Murray bifurcation fractal law and accurate quantification of lumen morphology by artificial intelligence algorithm [10]. Although theoretically, multiangle angiography has the potential to provide a more accurate description of lumen morphology, the anatomical features of the renal artery make the suboptimal angiography view (non-anterior-posterior position) often shortened and overlapped [13]. Therefore, the suboptimal angiographic view is redundant and may introduce additional errors.

Although our investigation showed that rQFR and rFFR had a decent level of concordance, minor inconsistencies were seen in some patients. The deviation could have a number of justifications. Firstly, the length of the vessel segment as determined by the pressure wire was longer than that which was calculated by the QFR. Due to insignificant stenoses, there may be a slight consumption of flow reserve in the investigated vessel. Additionally, highly inadequate intravascular contrast agent loading has the potential to hypothetically skew QFR calculations. Furthermore, inaccurate boundary condition estimates may also be responsible for the discrepancies.

Besides that, two recent prospective studies that assessed the predictive value of rFFR in predicting blood pressure improvement in patients receiving PTRAS revealed conflicting findings [6, 16, 17]. Kadziela et al. were unable to relate rFFR to an improvement in blood pressure or renal function [6], while Mitchell et al. demonstrated that rFFR is a viable method to identify patients likely to benefit from PTRAS [16, 17]. Both investigations used papaverine as a hyperemic agent and adhered to a cut-off value of 0.8, which is similar to the cut-off value used to define hemodynamically significant stenosis in the coronary artery [6], whereas Manoharan et al. demonstrated that papaverine causes an increase in average peak flow velocity that is roughly half as high as the rise observed following dopamine [18]. Therefore, it is plausible that the results of these experiments may have been affected by the inability to attain peak hyperemia [4, 19]. As such, a lower vasodilation level of renal vascular bed means that for a given stenosis. The determination of rQFR does not require the use of a pressure wire or induction of hyperemia and is based on angiography and Murray's bifurcation fractal law [20, 21]. It means that rQFR avoided the controversy over the choice of vasodilators and explains why rFFR got a lower value than rQFR in the vast majority of cases. Therefore, there are reasons to believe that rQFR would be more accurate at predicting outcomes than rFFR. Meanwhile, the presence of distinct differences between myocardial and renal physiology implies that successful FFR cut-off value in coronary interventions may not be fully transferable to the setting of renal artery stenosis, and further investigations of optimal cut-off of rQFR and rFFR are worthy and needed.

Moreover, the rapid QRA analysis based on automatic delineation of vessel contour generated by artificial intelligence algorithm of QFR software can not only accurately quantify the lesion morphology but also guide the selection of stent size during PTRAS. Besides, convenient intraoperative immediate assessment could realize the evaluation of hemodynamics after stent revascularization [20]. In our study, the rQFR of patients after revascularization increased to above 0.95.

Our study indicates that rQFR is feasible; however, successful rQFR measurement requires high-quality angiography and depends on a number of factors, including the following: (1) high-quality angiographic images for accurate rQFR measurement; (2) stable contrast medium injection speed during angiography; (3) for renal artery proximal lesion, excessive insertion of the catheter should be avoided; and (4) for severely twisted, dilated, or ostial renal artery lesion, rQFR measurement should be avoided.

In conclusion, rQFR may have potential clinical value in identifying ARAS with hemodynamic relevance and specific patient subgroups who may benefit from PTRAS as a novel renal artery hemodynamic parameter. The results of this study expand the application of QFR in catheterization laboratory. To continuously enhance the QFR calculation method used in peripheral artery interventional therapy, more prospective researches are needed.

## 5. Study Limitations

Obviously, there were certain limitations on our study. (1) In our study, the antihypertensive efficacy of PTRAS was evaluated using office BP. However, current international guidelines recommend out-of-office BP monitoring in clinical practice. Previous study showed the disproportional serial reductions between office and home SBP after PTRAS [22]. We believe that the criteria for assessing the antihypertensive efficacy of PTRAS are still open to discussion and debate. (2) Even though the rQFR suggests a therapeutic benefit for enhancing PTRAS outcomes, this needs to be evaluated in prospective trials. (3) Due to the retrospective study design, therapeutic strategies of enrolled patients were not based on a QRA assessment of the lesion, but rather on visual assessment during angiography; still, in all patients enrolled, QRA has been performed as part of our study protocol and diameter stenosis rate ranged from 50% to 90%. (4) To assess the correlation and consistency of rQFR in this pilot investigation, there was only a tiny validation set of rFFR data available. (5) The optimal cut-off rQFR value for predicting blood pressure improvement after PTRAS should be verified in larger prospective study.

## 6. Conclusions

Our study evaluated the clinical utility of rQFR assessment in ARAS patients, and rQFR bears the potential of improving angiography-based identification of hemodynamically significant renal artery stenosis during angiography and achieved comparable results to the invasive rFFR. However, larger and prospective investigations are required to corroborate our findings. To show the precise clinical significance of rQFR for ARAS patients, more research is necessary.

## Figures and Tables

**Table 1:** The clinical data of patients underwent optimal antihypertensive medication and PTRAS.

Variable	Revascularization (*n* = 37)	Optimized antihypertensive medications (*n* = 18)	*p* value
Age (years)	58.92 ± 10.30	51.72 ± 12.79	0.029
Body weight (kg)	66.43 ± 9.37	67.22 ± 7.00	0.753
Body height (cm)	166.19 ± 9.43	169.06 ± 8.50	0.280
Body mass index (kg/m^2^)	24.10 ± 3.21	23.62 ± 3.00	0.605
Female gender, *n* (%)	11 (29.72)	7 (38.89)	0.497
DM, *n* (%)	12 (32.43)	5 (27.78)	0.726
Previous CAD, *n* (%)	10 (27.02)	6 (33.33)	0.629
Previous stroke, *n* (%)	3 (8.10)	0 (0)	0.543
Smoking, *n* (%)	27 (72.98)	10 (55.56)	0.196
Systolic blood pressure (mmHg)	167.41 ± 11.03	160.17 ± 7.00	0.005
Diastolic blood pressure (mmHg)	95.41 ± 13.33	90.61 ± 7.23	0.161
Heart rate (beats per minute)	77.11 ± 14.48	73.61 ± 12.89	0.388
Creatinine^a^	93.68 ± 22.18	89.19 ± 19.76	0.470
Total cholesterol	4.47 ± 1.04	4.86 ± 1.42	0.310
Low-density lipoprotein cholesterol	2.59 ± 0.75	2.56 ± 0.78	0.877
Direct renin concentration^a^	110.58 (85.11-164.48)	45.50 (25.23-81.02)	<0.001
Related artery			
Left renal artery, *n* (%)	25 (67.67)	10 (55.56)	0.385
Right renal artery, *n* (%)	12 (32.43)	8 (44.44)	0.385
QRA^a^	72.00 (65.50-78.00)	65.00 (56.00-68.25)	0.001
rQFR	0.70 ± 0.15	0.79 ± 0.10	0.013

Abbreviations: DM: diabetes mellitus; CAD: coronary artery disease; QRA: quantitative renal angiography; rQFR: renal quantitative flow ratio. ^a^Comparison was made using the Mann–Whitney *U* test, and these values are expressed as median with interquartile range (25th and 75th percentiles).

**Table 2:** The clinical data of responders and nonresponders.

Variable	Responders (*n* = 25)	Non-responders (*n* = 12)	*p* value
Age (years)	57.08 ± 10.18	62.75 ± 9.87	0.118
Body weight (kg)	66.04 ± 9.22	67.25 ± 10.05	0.719
Body height (cm)	164.60 ± 9.39	169.50 ± 8.99	0.141
Body mass index (kg/m^2^)	24.42 ± 3.27	23.42 ± 3.12	0.383
Female gender, *n* (%)	9 (36.00)	2 (16.67)	0.279
DM, *n* (%)	9 (36.00)	3 (25.00)	0.711
Previous CAD, *n* (%)	6 (24.00)	4 (33.33)	0.696
Previous stroke, *n* (%)	2 (8.00)	1 (8.33)	>0.999
Smoking, *n* (%)	20 (80.00)	7 (58.33)	0.240
Systolic blood pressure (mmHg)	168.88 ± 12.18	164.33 ± 7.72	0.178
Diastolic blood pressure (mmHg)	97.76 ± 15.05	90.50 ± 6.96	0.122
Heart rate (beats per minute)	75.32 ± 14.92	80.83 ± 13.32	0.284
Creatinine^a^	90.77 ± 22.18	99.73 ± 21.85	0.265
Total cholesterol	4.70 ± 0.93	4.00 ± 1.14	0.057
Low-density lipoprotein cholesterol	2.73 ± 0.75	2.30 ± 0.68	0.096
Direct renin concentration^a^	128.93 (98.22-192.86)	87.17 (51.35-97.40)	0.001
Related artery			
Left renal artery, *n* (%)	19	6	0.146
Right renal artery, *n* (%)	6	6	0.146
No. of antihypertensive medications^b^	2.60 ± 0.87	3.00 ± 0.85	0.664
Application diuretics, *n* (%)^c^	4 (16.00)	6 (50.00)	0.049
QRA^a^	76.00 (66.50-79.00)	66.50 (61.00-69.75)	0.002
rQFR	0.64 ± 0.13	0.83 ± 0.06	<0.001

Abbreviations: DM: diabetes mellitus; CAD: coronary artery disease; QRA: quantitative renal angiography; rQFR: renal quantitative flow ratio. ^a^Comparison was made using the Mann–Whitney *U* test, and these values are expressed as median with interquartile range (25th and 75th percentiles). ^b^The numbers of antihypertensive medications taken by responder and nonresponder at 90 days following discharged from the hospital. ^c^The application of diuretics in responder and nonresponder at 90 days following discharged from the hospital.

## Data Availability

The data used to support the findings of this study are included in the article. Further requests for data, after publication of this article, can be directed to the corresponding author (xiaomeili1396@hotmail.com).
